# Safety and tolerability of artesunate-amodiaquine, artemether-lumefantrine and quinine plus clindamycin in the treatment of uncomplicated *Plasmodium falciparum* malaria in Kinshasa, the Democratic Republic of the Congo

**DOI:** 10.1371/journal.pone.0222379

**Published:** 2019-09-17

**Authors:** Yves Lula Ntamba, Hypolite Muhindo Mavoko, Marion Kalabuanga, Blaise Fungula, Pierre-Michel Ntamabyaliro Nsengi, Gaston Tona Lutete, Raquel Inocencio da Luz, Jean-Pierre Van geertruyden, Pascal Lutumba

**Affiliations:** 1 Clinical Pharmacology and Pharmacovigilance Unit, University of Kinshasa, Kinshasa, Democratic Republic of the Congo; 2 Global Health Institute, Faculty of Medicine, University of Antwerp, Antwerp, Belgium; 3 Department of Tropical Medicine, University of Kinshasa, Kinshasa, Democratic Republic of the Congo; 4 Lisungi Health Centre, Kinshasa, Democratic Republic of the Congo; Universidad Peruana Cayetano Heredia, PERU

## Abstract

**Introduction:**

Artemisinin-based combination therapy is currently the best option for the treatment of uncomplicated malaria. Quinine is recommended as a rescue treatment. Safety information during repeated treatment with the same drug is scarce. We report safety data from the Quinact randomized clinical trial (RCT) that was designed to assess efficacy and safety of artesunate-amodiaquine (ASAQ), artemether-lumefantrine (AL) and quinine+clindamycin (QnC).

**Methodology:**

Males and females aged 12 to 59 months with uncomplicated malaria were treated with ASAQ and followed up during 42 days (preRCT). Clinical failures were randomized to one of the 3 treatments and followed up for 28 days (RCT). Subsequent failures were repeatedly treated with ASAQ several times as needed (postRCT1, postRCT2 and so on) until a 28-days follow up period without parasitaemia.

**Results:**

Eight hundred and sixty-five, 242 and 64 patients were recruited respectively in preRCT, RCT and postRCTs. In preRCT, 433 (50.0%) patients experienced at least one drug-related adverse event (AE). The most reported AEs were anorexia (22.9%), asthenia (19.4%), and abnormal behavior (14.6%). Twenty-nine AEs (3.5%) were reported to be severe. In RCT, at least one drug-related AE was reported in 54.7%, 21.5% and 40.0% of patient randomized respectively to ASAQ, AL and QnC (p<0.001). During postRCT1 (n = 64), postRCT 2 (n = 17) and postRCT3 (n = 7), respectively 32.8%, 35.3% and 71.4% of patients experienced at least one drug-related AE. Three serious adverse events occurred but not judged related to study medication.

**Conclusion:**

The proportion of AEs did not increase over the treatment courses with ASAQ. However, continuous monitoring is important.

## Introduction

Artemisinin-based combination therapies (ACTs) are currently regarded as the best available choice for uncomplicated malaria treatment in accordance with the World Health Organization (WHO) guidelines. However, old drugs namely Quinine or Sulfadoxine plus Pyrimethamine (SP) are still used respectively as rescue treatment for uncomplicated malaria or intermittent preventive treatment in pregnancy in many countries [1]. In 2005, the National Malaria Control Programme (NMCP) of the Democratic Republic of Congo (DRC) adopted artesunate-amodiaquine (ASAQ), and a few years later added artemether-lumefantrine (AL) as first line regimens for uncomplicated malaria [2]. Implementation and use of these new combinations on a large scale make surveillance imperative. Besides, comparing to high consumption of antimalarial medicines in endemic countries, there is a dearth of published literature about ACT-induced adverse events (AEs) particularly in Africa. The published data are mainly from clinical trials with a classic design of limited number of participants, strict inclusion criteria or limited follow up [3–4]. However, information about ACT use in real-life settings is scarce [5]. In resource-limited settings especially in Sub-Saharan Africa (SSA), malaria control programmes face particular challenges concerning this surveillance. Pharmacovigilance systems in SSA are either non-existent, just growing or barely functional, sometimes not well or systematically structured, resulting in poor reporting [6]. This fact was emphasized in a recent report which outlined the lack of consistent inclusion of pharmacovigilance activities and requested funding support in proposals and in-country plans [7]. Lack of resources, infrastructure and expertise are the main reasons for the slow development of pharmacovigilance systems in developing countries [8–9]. In addition, weak drug regulatory authorities, counterfeit medicines, widespread use of herbal medicines and over-the-counter medications can really impend strategies addressed to implement pharmacovigilance system [6–7, 10–11]. Therefore, gathering information on the safety and tolerability of ACTs can be challenging [12]. Furthermore, ACTs’ choice in SSA was mainly based on the efficacy and safety data of a single malaria episode treatment, the impact of consecutive treatment courses with ACT on safety profile is not adequately known at study time period [10]. This is more relevant considering that in real life, people may use one drug repeatedly for presumed malaria attacks, especially as inappropriate use of antimalarials is a concern in Africa [13].

To bridge these gaps, data collected in the frame of the Quinact trial [14] conducted in the DRC allowed to assess the safety of ASAQ as first line treatment, then ASAQ, AL and oral Quinine plus clindamycin (QnC) when used as rescue treatment. Furthermore, ASAQ safety for repeated recurrent uncomplicated malaria episodes was assessed.

## Materials and methods

### Study site

The Quinact was a bi-centre clinical trial conducted in Kinshasa, DRC and Kazo, Uganda, from August 2012 (May 2012 for Uganda site) to April 2014. However, for completeness and consistency reasons, only data from the DRC site will be reported in this manuscript. The DRC is a malaria endemic country, where transmission is stable almost all over the country. The trial was conducted in the health zone of Mont-Ngafula 1, where asymptomatic *Plasmodium* infection is reported to be 23.1% in children under 5 years old [15]. Patients were recruited at Lisungi Health Centre.

### Study design

This was a phase IIIb, randomized, open label, three-arm trial, performed in three phases involving children aged from 12 to 59 months diagnosed with uncomplicated malaria. The trial was registered in ClinTrials.gov (NCT01374581) and the Pan African Clinical Trials Registry (PACTR201203000351114). The present manuscript focuses exclusively on safety aspects of all phases to a great extent including descriptive and comparative analyses. Efficacy findings are reported in the separate papers including brief descriptive safety data only during randomized phase [16–17]. Details concerning the study design and procedure are available in the published protocol [14].

#### First phase (pre-randomized controlled trial phase, pre RCT)

Patients having fulfilled recruitment criteria constituted a cohort that was treated with ASAQ and passively followed up for 42 days. In case of a clinical failure patients were eligible for the second phase. Subjects with known hypersensitivity or serious drug-related AE to the study drugs and the presence of significant underlying disease or condition were excluded in the study.

#### Second phase (randomized controlled trial phase, RCT)

Patients who experienced clinical failure during the first phase follow up, were randomly assigned to one of the 3 rescue treatments: either retreatment with ASAQ or AL (as an alternative ACT) or QnC, following a 2:2:1 ratio. Then they were actively followed up for 28 days, with a classic visit schedule for clinical and parasitological assessment [18]. Subjects with known hypersensitivity or serious drug-related AE to the study drugs were excluded in the study.

#### Third phase (post-randomized controlled trial phase, post RCTs)

Patients with clinical or parasitological failure from day 14 onwards were retreated with ASAQ and actively followed up following the same procedure as RCT phase. All subsequent failures were retreated with ASAQ several times as needed (postRCT1, postRCT2 and so on) until each patient was followed during 28 days without parasitaemia. The liver function was clinically monitored from the third ASAQ treatment course, because of the known hepatotoxicity of amodiaquine [19]. Laboratory assessment was performed based on clinical signs.

#### Safety endpoints

At each visit, AEs (clinical and biological if available) were recorded on the specific case report form (CRF) during the study period. Site investigators were trained for AE collection and assessment. Laboratory tests (haemoglobin, aspartate and alanine aminotransferase) were monitored on basis of worsened clinical signs of either anaemia or hepatotoxicity.

The AEs were assessed for causality using the WHO criteria [20]: certain (plausible time relationship to drug intake, cannot be explained by disease or other drugs, response to withdrawal plausible, rechallenge satisfactory, if necessary), probable (reasonable time relationship to drug intake, unlikely to be attributed to disease or other drugs, response to withdrawal clinically reasonable), possible (reasonable time relationship to drug intake, could also be explained by disease or other drugs) and unlikely (with a time to drug intake that makes a relationship improbable, disease or other drugs provide plausible explanations). The severity of a clinical AE was scored according to the following scale: mild (awareness of sign or symptom, but easily tolerated), moderate (discomfort enough to cause interference with usual activity), severe (incapacitating with inability to work or perform usual activity), life-threatening (patient at risk of death at the time of the event). International Conference for Harmonisation (ICH) definitions were used to assess AE seriousness [21]. AEs were considered as serious if: resulted in death or persistent disability, life threatened, required hospitalization or prolonged existing hospitalization, related to other medically important condition. At study completion, AEs were grouped according to the WHO adverse reaction terminology (WHO-ART) as System Organ Class (SOC) and Preferred Terms (PT) [22]. Only AEs with at least a “possible” relationship to drug were captured in this manuscript.

#### Study medication

During the first phase (Pre RCT), all subjects received ASAQ. Then those who experienced a treatment failure were randomly assigned to receive either ASAQ, AL or QnC (RCT phase). Thereafter all subsequent failures were retreated with ASAQ (Post RCT phase).

Treatment intake was directly observed by the study nurses, for all doses and all study phases. The drugs used in this trial were quality assured and storage conditions were regularly monitored. AL (Novartis, Basel, Switzerland) was administered twice daily for 3 days. Tablets contained 20 mg of artemether and 120 mg of lumefantrine. They were administered with a cup of milk (to improve lumefantrine absorption). Dosage was assigned according to manufacturer instructions: 5 to 14.9 kg, 1 tablet; 15 to 24.9 kg, 2 tablets; 25 to 34.9 kg, 3 tablets.

ASAQ (Sanofi, Casablanca, Morocco) was administered once daily for 3 days. Tablets contained 50 mg of artesunate and 135 mg of amodiaquine for patients weighing 9 to 17.9 kg; 100 mg of artesunate and 270 mg of amodiaquine for those weighing 18 to 35.9 kg.

Quinine (Sanofi, Gentilly, France) was administered thrice daily for 7 days. Tablets of 125 mg were available. Half of tablet was administered to patients weighing 9 to 11.9 kg, 1 tablet to those weighing 12 to 19.9 kg and 1.5 tablets to those weighing 20 to 27.9 kg.

Clindamycin (Pfizer SA/ NV Brussels, Belgium), granule for oral suspension 75mg/ 5 ml, was administered twice daily for at least 5 days at 10 mg/kg.

### Ethical considerations

The study was conducted in agreement with the principles of the “Declaration of Helsinki” and in accordance with the standards and codes of conduct accepted by the ICH guidelines. The study protocol was approved by the Committee for Medical Ethics of the Antwerp University Hospital, Belgium and the Ethical Committee of the School of Public Health, Kinshasa University, DRC. A first informed consent was signed or thumb-printed by parent/ legal guardian before enrolment in preRCT phase and a second one before enrolment in RCT phase. The last was written so as to also cover postRCT phase.

### Sample size and statistical analysis

The sample size was calculated on the basis of the RCT phase. Recruitment in the pre RCT phase was to be continued until the number of failures required for the randomized phase was reached [14]. The number (and percentage) of patients experiencing any AE was compared between treatment groups using Fisher's exact test. Data analysis was performed using STATA 12 statistical software packages (Stata Corp, Lakeway, College Station, Texas, USA). Categorical variables were compared between the treatment groups using chi-square tests or Fisher’s exact tests, and continuous variables were compared using t test. A p value of <0.05 was considered statistically significant. Univariate analysis using logistic regression was performed to identify the factors associated with occurrence of AEs and the strength of association. Factors with a p≤0.020 in univariate analysis were included in a stepwise multivariate modelling exercise using forward selection.

## Results

### Patient’s characteristics at inclusion

**Fig 1:**
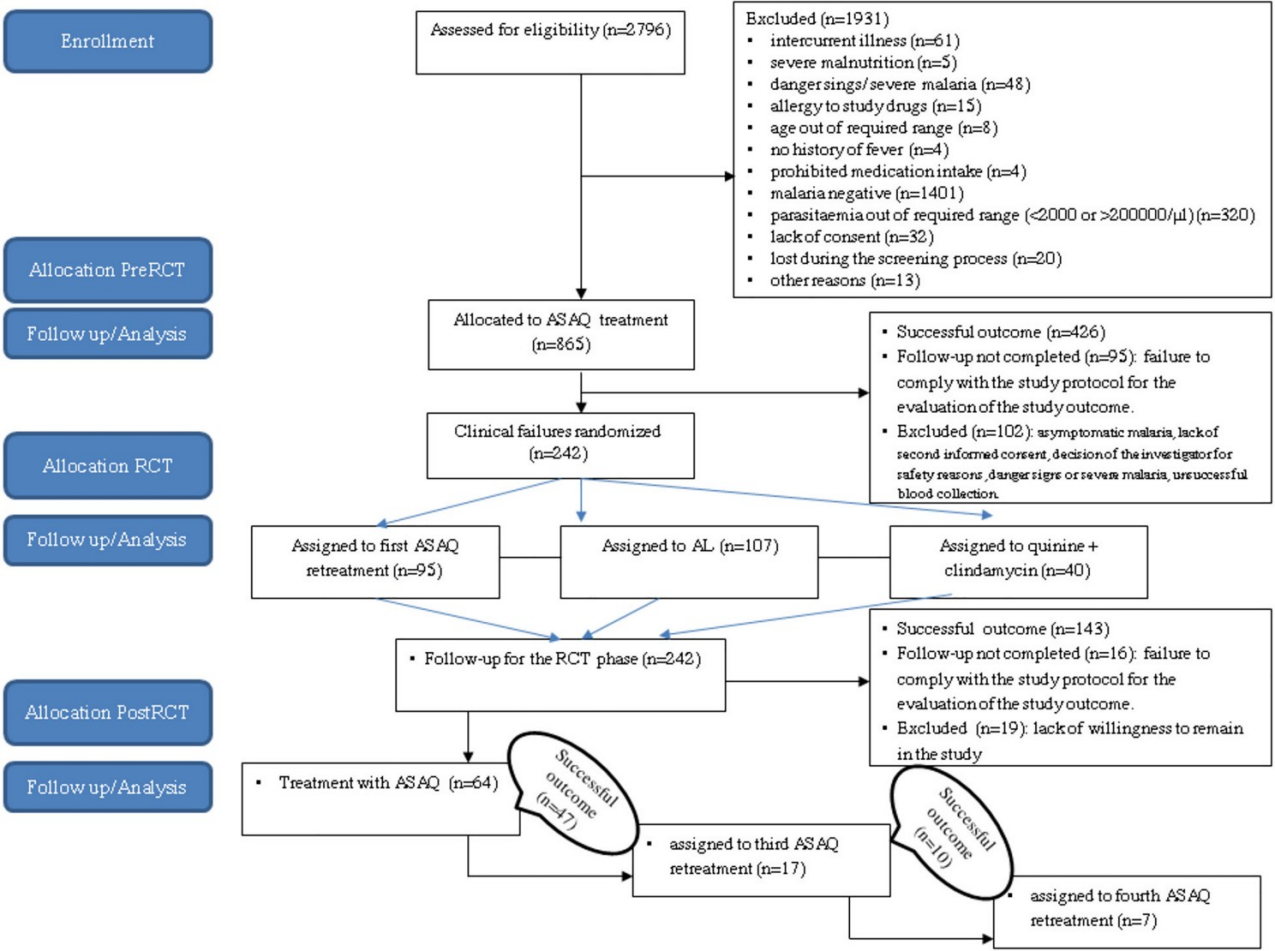
Trial flow chart, patients’ randomization and treatment assignment in Lisungi, Kinshasa, DR Congo.

In the pre RCT, 865 patients were enrolled and received ASAQ. This gave at least 50% of the chance of observing very common (occurring in 1 out of 10 patients taking the medicine), common (occurring at least in 1 out of 100 patients) and uncommon (occurring at least in 1 out of 1000 patients) adverse events. However, this phase offered little chance (less than 10%) to capture rare and very rare events [23–24]. Among clinical failures, 242 patients were enrolled in RCT phase. They were randomly assigned a rescue treatment as followed: 95 to ASAQ, to 107 to AL and 40 to QnC. Then 64, 17 and 7 were enrolled respectively in post RCT 1, 2 and 3, and they were always treated by ASAQ (Table 1, Fig 1).

**Table 1:** Baseline characteristics of patients enrolled in quinact trial (Kinshasa, DRC, 2012–14).

	Pre RCT n = 865	RCT n = 242	PostRCT1 n = 64	PostRCT2 n = 17	PostRCT3 n = 7
Gender, males, n (%)	442 (51.1)	129 (53.3)	31 (48.4)	12 (70.6)	7 (100)
Age (months), mean (SD)	36.2 (13.2)	37.4 (13.1)	37.4 (13.6)	35.7 (14.2)	28.9 (11.4)
Weight (kg), median (IQR)	12.5 (3.7)	13.0 (3.6)	12.9 (3.4)	12.9 (2.5)	13.3 (5.3)
Haemoglobin, mean (SD)	9.3 (1.6)	10.3 (1.6)	10.9 (1.2)	11.6 (1.6)	11.2 (0.7)

### Safety and tolerability during pre RCT

Of 865 patients recruited, 433 (50.0%) experienced at least one drug-related AEs. About one fourth (n = 229) exhibit two AEs or more. A total of 840 AEs was captured. The following systems were mainly involved in more than 30 AEs: psychiatric (38.6%, anorexia and abnormal behavior), gastro-intestinal (22.4%, especially abdominal pain and diarrhoea), general (20.5%, mainly asthenia), respiratory (12.5%, coughing in majority) and skin (3.8%, pruritus) (Table 2). The most reported AEs (more than 1%) were: anorexia (22.9%), asthenia (19.4%), abnormal behavior (including difficulty of concentration, agitation or confusion, 14.6%), coughing (11.3%), abdominal pain (10.2%), diarrhoea (5.7%), pruritus (3.4%), nausea (3.0%) and vomiting (2.9%) (Table 3). One case of either bullous eruption (verbatim “bullous eruption on lips”) or abnormal urine (verbatim “dark urine”) was reported, when 4 (0.5%) and 2 (0.2%) patients developed jaundice and hepatomegaly respectively. One AE (asthenia) led to drug discontinuation. Twenty-nine (3.5%) AE were graded as severe (Table 4). Three serious adverse events (SAE) were reported during pre RCT phase, 3 hospitalizations of which one resulted in death due to respiratory distress. None of them was judged to be related to the study medication (Table 5).

**Table 2:** Distribution of most relevant Adverse Event by treatment and phase study according Preferred Term (quinact trial, Kinshasa, DRC, 2012–14).

Phase	PreRCT (N = 865)	RCT (N, ASAQ = 95, AL = 107, QnC = 40)			PostRCT1 (N = 64)	PostRCT2 (N = 17)	PostRCT3 (N = 7)	Overall ASAQ
	ASAQ (n, %)	ASAQ (n, %)	AL (n, %)	QnC (n, %)	ASAQ (n, %)	ASAQ (n, %)	ASAQ (n, %)	ASAQ (n, %)
Total of patients (n)	865	95	107	40	64	17	7	1048*
**PT (WHO-ART)**								
**Abdominal pain**	88 (10.2)	7 (7.4)	6 (5.6)	1 (2.5)	4 (6.3)	4 (23.5)	1 (14.3)	104 (9.9)
**Abnormal behaviour**	126 (14.6)	10 (10.5)	2 (1.9)	6 (15.0)	8 (12.5)	3 (17.6)	5 (71.4)	152 (14.5)
**Anaemia**	5 (0.6)	0	0	1 (2.5)	0	0	0	5 (0.5)
**Anorexia**	198 (22.9)	24 (25.3)	6 (5.6)	8 (20.0)	13 (20.3)	3 (17.6)	5 (71.4)	243 (23.2)
**Asthenia**	168 (19.4)	14 (14.7)	2 (1.9)	6 (15.0)	13 (20.3)	3 (17.6)	5 (71.4)	203 (19.4)
**Bradycardia**	0	1 (1.0)	0	0	0	0	0	1 (0.1)
**Bullous eruption**	1 (0.1)	0	0	0	0	0	0	1 (0.1)
**Convulsions**	0	0	0	1 (2.5)	0	0	0	0
**Coughing**	98 (11.3)	16 (16.8)	6 (5.6)	0	4 (6.3)	4 (23.5)	3 (42.9)	125 (11.9)
**Diarrhoea**	49 (5.7)	7 (7.4)	7 (6.5)	1 (2.5)	3 (4.7)	4 (23.5)	1 (14.3)	64 (6.1)
**Dizziness**	2 (0.2)	0	1 (0.9)	0	0	0	0	2 (0.2)
**Hepatomegaly**	2 (0.2)	0	0	0	0	0	0	2 (0.2)
**Hypoglycaemia**	0	0	0	1 (2.5)	0	0	0	0
**Jaundice**	4 (0.5)	0	1 (0.9)	0	0	0	0	4 (0.4)
**Nausea**	26 (3.0)	3 (3.2)	1 (0.9)	1 (2.5)	0	1 (5.9)	0	30 (2.9)
**Oedema**	1 (0.1)	0	0	0	0	0	1 (14.3)	2 (0.2)
**Pruritus**	29 (3.4)	6 (6.3)	0	0	1 (1.6)	2 (11.8)	0	38 (3.6)
**Rash**	1 (0.1)	2 (2.1)	1 (0.9)	0	0	0	0	3 (0.3)
**Tachycardia**	2 (0.2)	0	1 (0.9)	2 (5.0)	0	0	0	2 (0.2)
**Tinnitus**	0	0	0	2 (5.0)	0	0	0	0
**Urine abnormal**	1 (0.1)	0	0	0	0	0	0	1 (0.1)
**Urticaria**	1 (0.1)	0	0	0	1 (1.6)	0	0	2 (0.2)
**Vomiting**	25 (2.9)	6 (6.3)	0	0	2 (3.1)	0	1 (14.3)	34 (3.2)

*number of treatment courses

**Table 3:** Distribution of Adverse Event by treatment and phase study according System Organ Class (SOC) (quinact trial, Kinshasa, DRC, 2012–14).

Phase	PreRCT (N = 865)	RCT (N, ASAQ = 95, AL = 107, QnC = 40)			PostRCT1 (N = 64)	PostRCT2 (N = 17)	PostRCT3 (N = 7)	Overall ASAQ
	ASAQ (n, %)	ASAQ (n, %)	AL (n, %)	QnC (n, %)	ASAQ (n, %)	ASAQ (n, %)	ASAQ (n, %)	ASAQ (n, %)
Total of AEs (n)	840	100	35	30	50	24	22	1036
**SOC**								
**General**	172 (20.5)	15 (15.0)	2 (5.7)	6 (20.0)	13 (26.0)	3 (12.5)	6 (27.3)	209 (20.2)
**Nervous**	3 (0.4)	0	1 (2.9)	1 (3.3)	0	0	0	3 (0.3)
**Gastro-intestinal**	188 (22.4)	23 (23.0)	14 (40.0)	3 (10.0)	9 (18.0)	9 (37.5)	3 (13.6)	232 (22.4)
**Hearing**	0	0	0	2 (6.7)	0	0	0	0
**Heart rate**	2 (0.2)	1 (1.0)	1 (2.9)	2 (6.7)	0	0	0	3 (0.3)
**Liver**	6 (0.7)	0	1 (2.9)	0	0	0	0	6 (0.6)
**Metabolic**	0	0	0	1 (3.3)	0	0	0	0
**Musculo-skeletal**	1 (0.1)	1 (1.0)	0	0	0	0	0	2 (0.2)
**Psychiatric**	324 (38.6)	34 (34.0)	8 (22.9)	14 (46.7)	21 (42.0)	6 (25.0)	10 (45.5)	395 (38.1)
**Red blood cell**	5 (0.6)	0	1 (2.9)	1 (3.3)	0	0	0	5 (0.5)
**Respiratory**	105 (12.5)	18 (18.0)	6 (17.1)	0	5 (10.0)	4 (16.7)	3 (13.6)	135 (13.0)
**Skin**	32 (3.8)	8 (8.0)	1 (2.9)	0	2 (4.0)	2 (8.3)	0	44 (4.2)
**Urinary**	1 (0.1)	0	0	0	0	0	0	1 (0.1)
**Vision**	1 (0.1)	0	0	0	0	0	0	1 (0.1)

**Table 4:** Distribution of AE by severity according Preferred Term (PT) (quinact trial, Kinshasa, DRC, 2012–14).

Total of events	PreRCT (n = 840)	RCT (n, ASAQ = 100, AL = 35, QnC = 30)			PostRCT1 (n = 50)	PostRCT2 (n = 24)
		ASAQ	AL	QnC		
	severe (n)	severe (n)	severe (n)	severe (n)	severe (n)	severe (n)
**PT (WHO-ART)**						
**Abnormal behaviour**	6	1	0	0	0	0
**Allergic reaction**	0	0	1	0	0	0
**Anaemia**	3	0	0	0	0	0
**Anorexia**	0	1	0	0	0	0
**Asthenia**	7	2	0	0	0	0
**Convulsions**	0	0	0	1	0	0
**Coughing**	9	1	2	0	2	1
**Fever**	2	0	0	0	0	0
**Pruritus**	1	0	0	0	0	0
**Rash**	0	0	1	0	0	0
**Urticaria**	1	0	0	0	1	0
**Total**	**29**	**5**	**4**	**1**	**3**	**1**

**Table 5:** Description of reported SAEs (quinact trial, Kinshasa, DRC, 2012–14).

Reactions	Received drug	Onset date	Relationship to drug	Age (month)	Gender	Co-morbidity	Outcome
**Respiratory distress**	ASAQ	Next day	Unlikely	31	F	Anemia, enteritis	Death
**Convulsion**	ASAQ	13^th^ day	Unlikely	54	F	Bacterial infection	Recovered
**Convulsion**	ASAQ	12^th^ day	Unlikely	44	M	None	Recovered

More than a half of subjects (n = 453, median weight 11.0 Kg IQR 10.0–12.0) received higher than ideal dose per body weight (>4mg/kg for artesunate and >10mg/kg for amodiaquine) without difference about gender. AEs occurred more frequently in those children and in patients who presented with fever while subjects with mixed infection at the recruitment exhibit less AEs regardless of analysis (uni- or multi-variate) (Table 6).

**Table 6:** Predictors of adverse events occurrence (quinact trial, Kinshasa, DRC, 2012–14).

	N = 865 n (%)	cOR (95% CI), p value	aOR (95% CI), p value
Predictors of AE occurrence			
Dosage, higher	453 (52.4)	1.48 (1.14–1.94), 0.004*	1.53 (1.17–2.01), 0.002*
Gender, male	441 (51.0)	0.93 (0.71–1.22), 0.61	NA
Anemia, hemoglobin < 11g/dl	729 (84.3)	1.03 (0.72–1.50), 0.84	NA
Patients with fever, ≥38°C	320 (37.0)	1.45 (1.10–1.92), 0.008*	1.47 (1.11–1.95), 0.007*
Patients sleeping under mosquito bednets the previous night	301 (34.8)	1.13 (0.86–1.50), 0.38	NA
Parasite infection, mixed	252 (29.1)	0.65 (0.48–0.88), 0.004*	0.64 (0.48–0.87), 0.004*

*statistically significant

### Safety and tolerability during RCT

At least one event was reported in 91 patients, 52 (54.7%), 23 (21.5%) and 16 (40.0%) respectively in ASAQ, AL and QnC arm. Of which 48 patients complained of AEs in Pre RCT phase too (30 received ASAQ, 10 AL and 8 QnC). Forty patients experienced two AEs or more. Overall 165 AE were reported, of which 100 in ASAQ group, 35 in AL group and 30 in QnC group. (Table 3). Ten AEs (6.1%) were graded as severe. No SAE was reported during this phase. Psychiatric disorders (46.7% and 34.0% in QnC or ASAQ arm, anorexia and abnormal behavior especially) were the most AE reported, followed by gastro-intestinal disorders (40.0% in AL arm, mainly abdominal pain, diarrhoea, nausea and vomiting), respiratory disorders (coughing particularly), general disorders (especially asthenia), skin disorders (mainly pruritus) (Tables 2 and 3). One case of hypoglycaemia was reported in QnC group. One case of each the following AE *id est* anaemia and convulsions, jaundice were reported respectively for QnC and AL. Significant differences arose from treatment groups, ASAQ tended to be more responsible of AE occurrence than other antimalarials except for general and hearing disorders where QnC was respectively more involved than AL (p = 0.002) and both AL (p = 0.020) and ASAQ (p = 0.028) (Table 7). High proportions were observed in ASAQ vs AL arm regarding general (p = 0.001), psychiatric (p <0.001) and skin disorders (p = 0.010) whereas compared to QnC, ASAQ induced more gastro-intestinal (p = 0.049) and respiratory disorders (p = 0.003) (Table 7).

**Table 7:** AE occurrence by treatment according SOC (RCT phase) (quinact trial, Kinshasa, DRC, 2012–14).

	RCT, Number of patients with AE (n = 91)					
	ASAQ (n = 52)	AL (n = 23)	QnC (n = 16)			
Patients allocated to treatment (N)	N = 95	N = 107	N = 40			
% of patients with at least 1 AE	54.7%	21.5%	40.0%			
SOC (WHO-ART)	ASAQ, n = 52 (%)	AL, n = 23 (%)	QnC, n = 16 (%)	p value		
				ASAQ vs AL	ASAQ vs QnC	AL vs QnC
**General**	13 (25%)	2 (8.7%)	6 (37.5%)	0.001*	0.841	0.002*
**Nervous**	0	1 (4.3%)	1 (6.3%)	0.345	0.122	0.466
**Gastro-intestinal**	17 (32.7%)	12 (52.2%)	2 (12.5%)	0.177	0.049*	0.253
**Hearing**	0	0	2 (12.5%)	**NA**	0.028*	0.020*
**Heart rate**	1 (1.9%)	1 (4.3%)	2 (12.5%)	0.933	0.155	0.121
**Liver**	0	1 (4.3%)	0	0.345	**NA**	0.54
**Metabolic**	0	0	1 (6.3%)	**NA**	0.122	0.101
**Musculo-skeletal**	1 (1.9%)	0	0	0.287	0.515	**NA**
**Psychiatric**	28 (53.8%)	8 (34.8%)	10 (62.5%)	<0.001*	0.598	0.004*
**Red blood cells**	0	1 (4.3%)	1 (6.3%)	0.345	0.122	0.466
**Respiratory**	18 (34.6%)	6 (26.1%)	0	0.003	0.003*	0.126
**Skin**	8 (15.4%)	1 (4.3%)	0	0.010*	0.058	0.54
Total	**52**	**23**	**16**			

*statistically significant

NA: not applicable

### Safety and tolerability during post RCT

#### Post-RCT1

Twenty-one (32.8%) out of 64 patients complained of at least one AE with a total of 50 AEs recorded. Eleven (17.2%) subjects complained of two or more AEs. Three patients experienced any AE with ASAQ during the 3 phases of study (Pre RCT, RCT and Post RCT1). From those AEs, 3 (6%) were severe. However, no SAE was accounted. Anorexia, asthenia and abnormal behavior were the commonest AE reported (> 5 reports) (Table 2). Psychiatric and general systems were the most affected (> 10 AE) (Table 3).

#### Post-RCT2

Of 17 patients treated, 6 (35.3%) experienced drug-related AE and 24 AEs were captured. Only 1 AE was assessed as severe. Abdominal pain, coughing and diarrhoea represented the half of reported AEs (Table 2). Gastro-intestinal and psychiatric systems were the most concerned (> 50% for both) (Table 3).

#### Post-RCT3

During the ultimate phase, 22 drug-related AE were accounted from 5 out of 7 patients treated (4.4 AE per patient). Neither severe AE nor SAE were noted. Abnormal behavior, anorexia and asthenia were most common when psychiatric and body as a whole-general system were mainly involved (Tables 2 and 3).

### Overall assessment

During the entire period of this study, respectively 1048, 107 and 40 treatment courses of ASAQ, AL and QnC were administered to all patients. Overall 1101 AEs were reported with the 3 antimalarial regimens of which 1036 ASAQ-induced AEs (0.99 AE per treatment), 35 AL-induced (0.33 AE per treatment) and 30 QnC-induced (0.75 AE per treatment). Forty-three AEs (3.9%) were graded as severe (Table 7). The most common system affected (respectively for ASAQ, AL and QnC) were psychiatric (38.1%, 22.9% and 46.7%), gastro-intestinal (22.4%, 40.0% and 10.0%), general (20.2%, 5.7% and 20.0%). The most reported AEs (respectively for ASAQ, AL and QnC) were anorexia (23.2%, 5.6% and 20.0%), asthenia (19.4%, 1.9% and 15.0%) and abnormal behavior (14.5%, 1.9% and 15.0%). Tables 2, 3 and 7 outline AEs classification (PT and SOC) according antimalarial regimen on the one hand and classification by severity on the other one.

## Discussion

In malaria endemic countries, ACTs are widely [18] and likely repeatedly used to treat recurrent episodes; though there is a necessity to assess their safety profile [25, 26]. The present findings show numerous related-antimalarial AEs but they were mostly minor. Nonetheless, some particular AEs had been noted. Abnormal behavior was common although the literature reports behavioral changes as rare AEs of 4-aminoquinoline derivatives (including amodiaquine) and the summary of product characteristics (SmPC) does not mention it among psychiatric disorders [27, 28]. One study about effect of administration of amodiaquine on rats highlighted various behavioral disorders due to drug effects [29]. However, it is not excluded that these (subjective) effects were over-reported considering the fact of participating in a clinical trial was a new experience for both parents and child. Caution may be needed to monitor closely these adverse effects while antimalarial amodiaquine-contained regimen is given. One case of bullous eruption was recorded after ASAQ administration while it is generally not reported in other studies [3, 30]. However, several cases had been captured with AL [31]. Abnormal urine (dark urine) could suggest haemoglobinuria and haemolysis with likely underlying enzymes deficiency (e.g. G6PD). This could be confirmed by laboratory analysis which was not assessed in this study. However, haemoglobinuria has been recorded in clinical trials with artemisinin derivatives (either artemether or artesunate) [32, 33]. For some events such as hepatomegaly, splenomegaly, conjunctivitis, it is unclear if they are related to drugs or occurred as a result of the underlying disease or condition (coincidental event) [27]. Hepatomegaly (graded as mild) reported here occurred simultaneously with asthenia or jaundice in 2 children (0.2%), they may all be prodromal clinical signs of drug induced-hepatotoxicity even if time of onset is relatively short (one day) [34]. In general, most of AEs were mild or moderate even during ultimate phase.

These findings are in line with other studies even when repeated treatment has been given [3, 10, 35]. ASAQ was also found responsible of significantly higher events rate than other antimalarials in the literature [3, 29]. However, one recent study shows that AEs were no more numerous in ASAQ group than in AL one, even in cases of readministration [36]. In one cohort, bradycardia has been also identified as a potential risk on the second day of treatment with Amodiaquine [37]. However, electrocardiogram was not performed in this study. Some AEs such as hypoglycaemia, severe asthenia, anaemia may need close monitoring to avoid threatened outcome or discontinuation of the therapy. The analysis indicated relationship between AE occurrence and the ASAQ dose. The dose-dependent effects (gastro-intestinal or neutropenia) of antimalarials (such as ASAQ or AL, artesunate monotherapy) were reported in other studies [38–40].

The findings of this study are mostly similar to other studies that also reported almost same AE pattern and are within the expected type based on the SmPC of the medications expect psychiatric disturbances [3, 27, 30, 41]. Nonetheless, this study was not powered to detect rare events (occurring in < 0.1% of the population treated). Some of antimalarial AEs such as nausea, vomiting, asthenia or anorexia are similar to malaria symptoms; making it often difficult to discriminate possible drug events from disease effects [42]. Moreover, anorexia is only considered as psychiatric disorder in the WHO-ART and includes “loss of appetite” as well as “decreased appetite”, when in other system such as Medical Dictionary for Regulatory Activities “MedDRA” terminology anorexia may fall under metabolism and nutrition disorder or psychiatric disorder categories; this could influence AE reporting following the system used [22, 43]. Nevertheless, this study provides useful information on safety profile of antimalarials especially when administered to young children.

Some limitations must be mentioned. Patients having previous history with serious adverse events were not included in the study; this may affect external validity. The unblinded design of the RCT may bias the result considering the differential reporting or recall between the groups. Electrocardiogram evaluations were not performed while ASAQ is known to be associated with risk of cardiotoxicity [44].

## Conclusion

This study indicated a good safety profile in case of repeated treatment, although the number of participants was decreasing over the study phases. However, safety monitoring should continue in routine clinical practice in the context of wide and extend use of ACTs, mainly in Africa. The study did not provide definitely evidence of safe biological profile, laboratory investigations schedule may be necessary but their implementation in the routine activities would be a challenge. Healthcare providers should be recommended to advice and encourage patients to complete their medications as the expected reactions are most often not severe.

## Supporting information

S1 File CONSORT 2010 checklist for quinact study safety paper. (DOC)
